# Prognostic Significance of Solid Subtype of Papillary Thyroid Carcinoma: Analysis of 63 Patients

**DOI:** 10.1002/hed.28266

**Published:** 2025-07-29

**Authors:** Andre Ywata de Carvalho, Felipe D' Almeida Costa, Jose Guilherme Vartanian, Luiz Paulo Kowalski

**Affiliations:** ^1^ Head and Neck Surgery and Otorhinolaryngology Department A. C. Camargo Cancer Center Sao Paulo Brazil; ^2^ Pathology Department A. C. Camargo Cancer Center Sao Paulo Brazil; ^3^ Head and Neck Surgery Department University of Sao Paulo Sao Paulo Brazil

**Keywords:** aggressive variant, papillary carcinoma, recurrence, solid subtype, solid trabecular variant, thyroid

## Abstract

**Objective:**

Solid subtype of papillary thyroid carcinoma (SSPTC) is an uncommon subtype of papillary thyroid cancer (PTC) and its prognostic significance is still controversial. The aim of this review is to present the clinicopathological features of SSPTC and compare its clinical behavior with classical PTC (cPTC).

**Methods:**

We retrospectively analyzed patients who underwent thyroidectomy for SSPTC and compared the clinicopathological findings and clinical outcomes with those of patients with cPTC.

**Results:**

Among 4937 patients treated for PTC, 63 (1.3%) were diagnosed with SSPTC, of which 76.2% were female, with a median age of 40 years, 3.2% had prior cervical irradiation, and the median diameter of the tumor was 15 mm. Compared to cPTCs, SSPTCs were larger (median tumor diameter: 15 vs. 9 mm), more symptomatic (non‐incidentally discovered) and were associated with an elevated risk for multifocality, extrathyroidal extension, lympho‐vascular invasion, lymph node metastasis, extra‐nodal extension, and distant metastasis. After a mean follow‐up of 58.5 months, rates of biochemical incomplete response to treatment and structural recurrence were higher when compared to those of cPTC. None of the patients who demonstrated an excellent response to treatment presented cancer recurrence during follow‐up. Distant metastasis occurred in 3 patients (4.8%). There were no cancer‐related deaths.

**Conclusions:**

SSPTC is an aggressive histological subtype of PTC associated with adverse prognostic features, including larger tumor size, multifocality, and extrathyroidal extension, which correlate with a higher risk of cancer recurrence and therefore should be considered for accurate risk stratification, comprehensive management, and close follow‐up.

**Abbreviations**
ATA: American Thyroid Association
CI: confidence interval
cPTC: classical papillary thyroid carcinoma
HR: hazard ratio
NIFTP: noninvasive follicular thyroid neoplasm with papillary‐like nuclear features
Ns‐Tg: nonstimulated thyroglobulin
pN1: lymph node metastasis pathologically confirmed
pT: pathological tumor
RAI: radioactive iodine
RFS: recurrence‐free survival
SD: standard deviation
SSPTC: solid subtype of papillary thyroid carcinoma
S‐Tg: stimulated thyroglobulin
Tg: thyroglobulin
TSH: thyroid‐stimulating hormone
TT: total thyroidectomy
WHO: World Health Organization

## 1 Introduction

The incidence of thyroid cancer is increasing worldwide, with papillary thyroid cancer (PTC), the most common and least aggressive histologic type, accounting for most of the new cases [1]. Excellent outcomes following therapy of PTC were demonstrated, with 10‐year survival rates of 93% [2]. However, despite the favorable long‐term prognosis, locoregional recurrences have been described in up to 28% of the patients [3] and the mortality rate is increasing, especially in advanced‐stage tumors [4].

Pathologic examination of thyroid surgical samples provides important information for risk stratification of cancer and postoperative patient management [5]. The new World Health Organization (WHO) classification uses “subtype” instead of “variant” in order to avoid confusion with genetic variants and to standardize the terminology across all the volumes of the fifth edition of the WHO classification [6]. Some of the PTC histologic subtypes are associated with more aggressive tumor behavior and unfavorable outcomes, as the tall cell, diffuse sclerosing, columnar cell, hobnail, and solid/trabecular subtypes [7].

First described in 1985 [8], the solid subtype of papillary thyroid carcinoma (SSPTC) accounts for 0.8%–3% of all subtypes of PTC [9, 10]. A higher proportion (34%) of SSPTC cases was observed among children exposed to radiation after the Chernobyl nuclear accident [11]. Regarding clinical outcomes following SSPTC treatment, there are conflicting results, as some authors suggest higher rates of distant metastasis, cancer recurrence, and mortality [10, 12] while others do not [13, 14].

In this study, we reviewed the clinicopathological features and clinical outcomes of SSPTC and compared them with cPTC.

## 2 Materials and Methods

### 2.1 Study Population and Treatment

After obtaining institutional review board approval (number 3055/21), we retrospectively reviewed the medical records of patients who underwent thyroidectomy for PTC between January 1996 and June 2017. Data from this cohort were retrospectively collected and continuously updated from patient records. The inclusion criteria were: (1) a postoperative pathological diagnosis of SSPTC or cPTC, and (2) availability of clinical and pathological data. The exclusion criteria included: the presence of concomitant thyroid malignancies (*n* = 21) and histological subtypes other than SSPTC or cPTC (*n* = 125). Of the 5083 patients initially identified, 4937 were included in this study. Missing data were noted in some patients, including response to treatment (888 patients; 18%), anti‐thyroglobulin antibody levels (460 patients; 9.3%), and postoperative stimulated thyroglobulin (s‐Tg) levels (1931 patients; 39.1%). Only patients with a postoperative pathologic diagnosis of SSPTC or cPTC were included, and patients with concurrent thyroidal malignancies were excluded. For patients who were pathologically confirmed to have high‐risk findings, mainly extrathyroidal extension and lymph node metastasis, routine radioactive iodine (RAI) treatment was administered. Thyroid‐stimulating hormone (TSH), postoperative stimulated thyroglobulin (s‐Tg) levels, and anti‐Tg antibody levels were measured before RAI, and a whole‐body scintigraphy was performed 2–5 days after RAI therapy.

### 2.2 Clinicopathological Features

Patient characteristics, treatment information, pathological features, and postoperative clinical outcomes were analyzed. Histologically, SSPTC was defined by a solid, trabecular, or nested cellular growth pattern, which represent more than 50% of the tumor and exhibits nuclear features typical of PTC [15]. Patients were classified according to the 2015 American Thyroid Association (ATA) risk stratification system as low, intermediate, or high risk for recurrence [5]. Histopathological diagnosis of SSPTC was based on the original pathology reports, and no systematic re‐examination of slides was performed.

### 2.3 Follow‐Up and Clinical Outcomes

Patients were assessed every 3 months for the first year, every 6 months between the second and fifth years, and every 12 months thereafter at the discretion of the attending physician based on the risk of the individual patient. The follow‐up visits included palpation of the neck, dosage of serum TSH, Tg, and anti‐Tg antibody levels, and ultrasound examination of the cervical lymph nodes. Patients were stratified according to biochemical response following therapy based on postoperative Tg levels during the first year of follow‐up: excellent response (ns, nonstimulated Tg ≤ 0.2 ng/mL following total thyroidectomy [TT] + RAI, ns‐Tg ≤ 1 ng/mL following TT without RAI or ns‐Tg ≤ 30 ng/mL following partial thyroidectomy, no rising levels of anti‐Tg antibodies); incomplete response (ns‐Tg ≥ 1 ng/mL following TT + RAI, ns‐Tg ≥ 5 ng/mL following TT without RAI or ns‐Tg > 30 ng/mL following partial thyroidectomy, rising anti‐Tg antibody levels) and indeterminate response (nonspecific biochemical results, which cannot be confidently classified as either excellent or incomplete response) [16]. Structural disease recurrence was defined as the reappearance of cancer following initial therapy. This included all events reported (local recurrences, lymph node metastases, and distant metastases) and confirmed by biopsy/surgery.

### 2.4 Statistical Methods

For statistical evaluation of differences between groups, the non‐parametric Mann–Whitney *U* test was applied. Categorical variables were analyzed using the chi‐square test. For 2 × 2 tables, Fisher's exact test was used when at least one expected frequency was less than 5. The Shapiro–Wilk test was applied to verify the normality of the data. The probabilities of disease‐free survival were estimated using the Kaplan–Meier technique, and the log rank test was applied to verify differences between the survival curves for each variable. The follow‐up time, in months, was calculated from the date of surgery to the date of death or the date of last information for censored cases and, for relapsed cases, the date of disease recurrence. The 5% significance level was considered for all statistical tests. The Cox proportional hazards model was performed to estimate the crude relative risks or crude hazard ratio (HR) and respective 95% confidence interval (95% CI) for recurrence events. The multivariate model provided the multivariate hazard ratio and respective 95% confidence interval. The retrospective selection technique was performed to choose the variables, and *p* < 0.10 was specified to select variables for the final model and thus to define potential independent prognostic factors for disease recurrence. Schoenfeld and scaled Schoenfeld residuals were calculated by checking whether the proportional hazards assumption is valid for the final multivariate Cox regression model. All statistical analyzes were performed using Stata software: version 16 (StataCorp LP, College Station, Texas).

## 3 Results

We analyzed a total of 4937 patients treated for papillary thyroid carcinoma, of which 63 (1.3%) were diagnosed with SSPTC.

**FIGURE 1:**
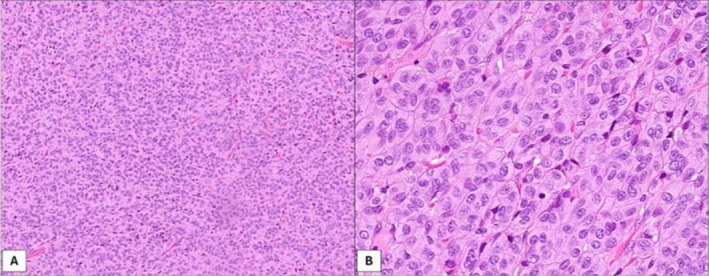
(A) Histologically, tumors show a solid, trabecular or nested growth pattern equivalent to at least 50% of the tumor mass; (B) the tumor cells present nuclear features characteristic of conventional PTC, including oval shape, irregular nuclear contours, ground‐glass nuclei, nuclear grooves, and pseudo‐inclusions. [Color figure can be viewed at wileyonlinelibrary.com]

Among SSPTC patients, 48 were women and 15 were men, with a sex ratio of 3:1. The median age of patients at diagnosis was 40 years (ranged from 15 to 76 years). Two of the 63 patients with SSPTC had a prior history of neck irradiation due to previous treatment for breast cancer and soft tissue sarcoma. SSPTCs ranged in size from 4 to 60 mm and the median tumor size was 15 mm. Multifocal tumors were observed in 30 patients (47.6%), of which 20 (31.8%) were bilateral. Most tumors were incidentally discovered and 19 (30.2%) patients presented symptoms at diagnosis: palpable nodule in 15 (23.8%), metastatic neck lymph nodes in 3 (4.8%), and hoarseness in 1 patient (1.6%). Microscopically, tumors showed a solid, trabecular, or nested growth pattern equivalent to at least 50% of the tumor mass (Figure 1A). In all cases, the tumor cells presented nuclear features characteristic of conventional PTC, including oval shape, irregular nuclear contours, ground‐glass nuclei, nuclear grooves, and pseudo‐inclusions (Figure 1B). Lymphatic invasion was observed in 10 cases (15.9%), vascular invasion in 6 cases (9.5%) and 3 cases (4.8%) had perineural invasion. Lymph node metastasis was pathologically confirmed postoperatively in 19 (30.2%) patients, of which 13 (68.4%) had extra‐nodal extension. Chronic lymphocytic thyroiditis was found in 27 cases (42.9%). Most patients were treated by total thyroidectomy (98.4%), without neck lymph node dissection (71.4%), followed by adjuvant radioactive iodine therapy (85.5%). According to the ATA risk stratification system [5], the majority (92.1%) of patients with SSPTC were classified as intermediate‐risk, and 62.5% of patients demonstrated an excellent response following treatment.

The clinicopathological characteristics, treatment‐related variables, and clinical outcomes of patients with SSPTC are presented in Table 1 and compared to cPTC.

**TABLE 1:** Comparison between solid and classical subtypes of papillary thyroid carcinoma according to clinicopathological characteristics, treatment‐related variables, and clinical outcomes.

		Solid subtype		*p*
		Yes	No	
		*N* (%)		
Total		63 (1.3)	4874 (98.7)	0.418
Male sex		15 (23.8)	961 (19.7)	
Age (years)	Mean ± SD ≥ 55 years	41.1 ± 14.2	43.7 ± 13.1	0.131*
		11 (17.5)	1052 (21.6)	0.429
Previous neck irradiation		2 (3.2)	62 (1.4)	0.215*
Non‐incidental diagnosis		19 (30.2)	779 (15.5)	0.001
Tumor size (mm)	Mean ± SD > 10 mm	17.4 ± 11.7	10.9 ± 9.3	< 0.001*
		49 (77.8)	2189 (44.9)	< 0.001
Multifocality		30 (47.6)	1620 (33.2)	0.016
Bilaterality		20 (31.8)	1103 (22.6)	0.087
Extrathyroidal extension	Minor	22 (34.9)	1084 (22.2)	0.001
	Gross	3 (4.8)	53 (1.1)	0.002
Vascular invasion		6 (9.5)	84 (1.7)	0.001*
Lymphatic invasion		10 (15.9)	107 (2.2)	< 0.001*
Chronic thyroiditis		27 (42.9)	1644 (33.7)	0.128
Lymph node metastasis pN1		19 (30.2)	889 (18.3)	0.015
Extra‐nodal extension		13 (20.6)	358 (7.4)	< 0.001
Total thyroidectomy		62 (98.4)	4810 (98.7)	0.568*
Neck dissection		18 (28.6)	858 (17.6)	0.024
Adjuvant RAI therapy		53 (85.5)	2929 (61.0)	< 0.001
Ps‐Tg > 10 ng/mL		11 (22.4)	432 (14.6)	0.125
Anti‐Tg antibodies		16 (26.7)	849 (19.2)	0.147
ATA risk category	Low	0	3251 (66.7)	NA
	Intermediate	58 (92.1)	1566 (32.1)	< 0.001
	High	5 (7.9)	56 (1.2)	< 0.001*
Clinical response to initial therapy classification	Excellent	35 (62.5)	3044 (76.2)	NA
	Incomplete	10 (17.9)	227 (5.7)	0.001
	Indeterminate	11 (19.6)	722 (18.1)	0.417
Distant metastasis		3 (4.8)	18 (0.4)	0.002*
Recurrence		8 (12.7)	208 (4.3)	0.006*
Time to recurrence (months)	Variation	4.7–57	1.1–160.9	0.012**
	Median	17.2	21.7	

*Note: p*‐value calculated using the Chi‐square frequency test.

Abbreviations: ATA, American Thyroid Association; NA, not assessable statistically; Ps‐Tg, postoperative stimulated thyroglobulin; RAI, radioactive iodine; SD, standard deviation; Tg, thyroglobulin.

* *p*‐value calculated using the Fisher exact test.

** *p*‐value calculated using the Mann–Whitney *U*‐test.

Compared to cPTCs, SSPTCs were larger, with a median tumor diameter of 15 mm (range, 4–60 mm) versus 9 mm (1–140 mm), more symptomatic (non‐incidentally discovered), and were associated with a higher risk of multifocality, extrathyroidal extension, lympho‐vascular invasion, pathologically confirmed lymph node metastasis, and extra‐nodal extension.

After a median follow‐up of 58.5 months (range, 10–298.9 months), structural tumor recurrence was diagnosed in 216 (4.4%) patients, of whom 26 (12%) patients had true cancer recurrences after excellent response to treatment and 190 (88%) patients had persistent cancer after incomplete or indeterminate biochemical response. Tumor recurrence was diagnosed in 208 (4.3%) patients with cPTC and in 8 (12.7%) patients with SSPTC. Among the 8 patients with SSPTC who experienced recurrence, 5 (62.5%) had regional (lymph node) recurrence and 3 (37.5%) had distant metastases (lung in 2 patients and bone in 1). There were no cancer‐related deaths. Incomplete biochemical response to treatment, locoregional recurrence, and distant metastases were more frequent in patients with SSPTC compared to patients with cPTC. Cancer recurrence was observed in 1 of the 11 SSPTC patients who demonstrated an indeterminate response to treatment, and none of the 35 patients with an excellent response experienced recurrence during follow‐up. In univariate, but not in multivariate Cox regression analysis, SSPTC was associated with cancer recurrence (Table 2).

**TABLE 2:** Univariate and multivariate analysis of risk of recurrence in patients with papillary thyroid carcinoma.

		Univariate analysis		*p* **	Multivariate analysis		*p* **
		HR*	95% CI*		HR*	95% CI*	
Male sex		1.6	1.19–2.15	0.002			
Age < 55 years		1.88	1.28–2.76	0.001	1.56	1.06–2.31	0.025
Previous neck irradiation		1.14	0.42–3.07	0.790			
Non‐incidental diagnosis		1.83	1.34–2.48	< 0.001			
Tumor size > 10 mm		2.56	1.96–3.36	< 0.001	1.54	1.15–2.05	0.003
Multifocality		1.89	1.45–2.45	< 0.001			
Bilaterality		1.6	1.21–2.12	0.001			
Solid subtype		2.57	1.27–5.22	0.009			
Extrathyroidal extension	Minor	2.36	1.78–3.12	< 0.001	1.34	1.0–1.81	0.052
	Gross	9.4	5.66–5.61	< 0.001	3.43	1.99–5.91	< 0.001
Vascular invasion		2.84	1.62–4.99	< 0.001			
Lymphatic invasion		2.76	1.66–4.6	< 0.001			
Chronic thyroiditis		0.83	0.63–1.11	0.21			
pT	1b	1.65	1.14–2.4	0.008			
	2	2.1	1.24–3.56	0.006			
	3	3.22	2.32–4.46	< 0.001			
	4	10.95	6.45–18.58	< 0.001			
pN1		5.77	4.43–7.52	< 0.001	4.03	2.98–5.43	< 0.001
Adjuvant RAI therapy		3.85	2.62–5.66	< 0.001			

Abbreviations: 95% CI, 95% confidence interval; HR, hazard ratio; pN1, lymph node metastasis pathologically confirmed; Ps‐Tg, postoperative stimulated thyroglobulin; pT, pathological tumor; RAI, radioactive iodine.

* HR and 95% CI estimated by Cox regression models.

** *p*‐value calculated using the log‐rank test.

**FIGURE 2:**
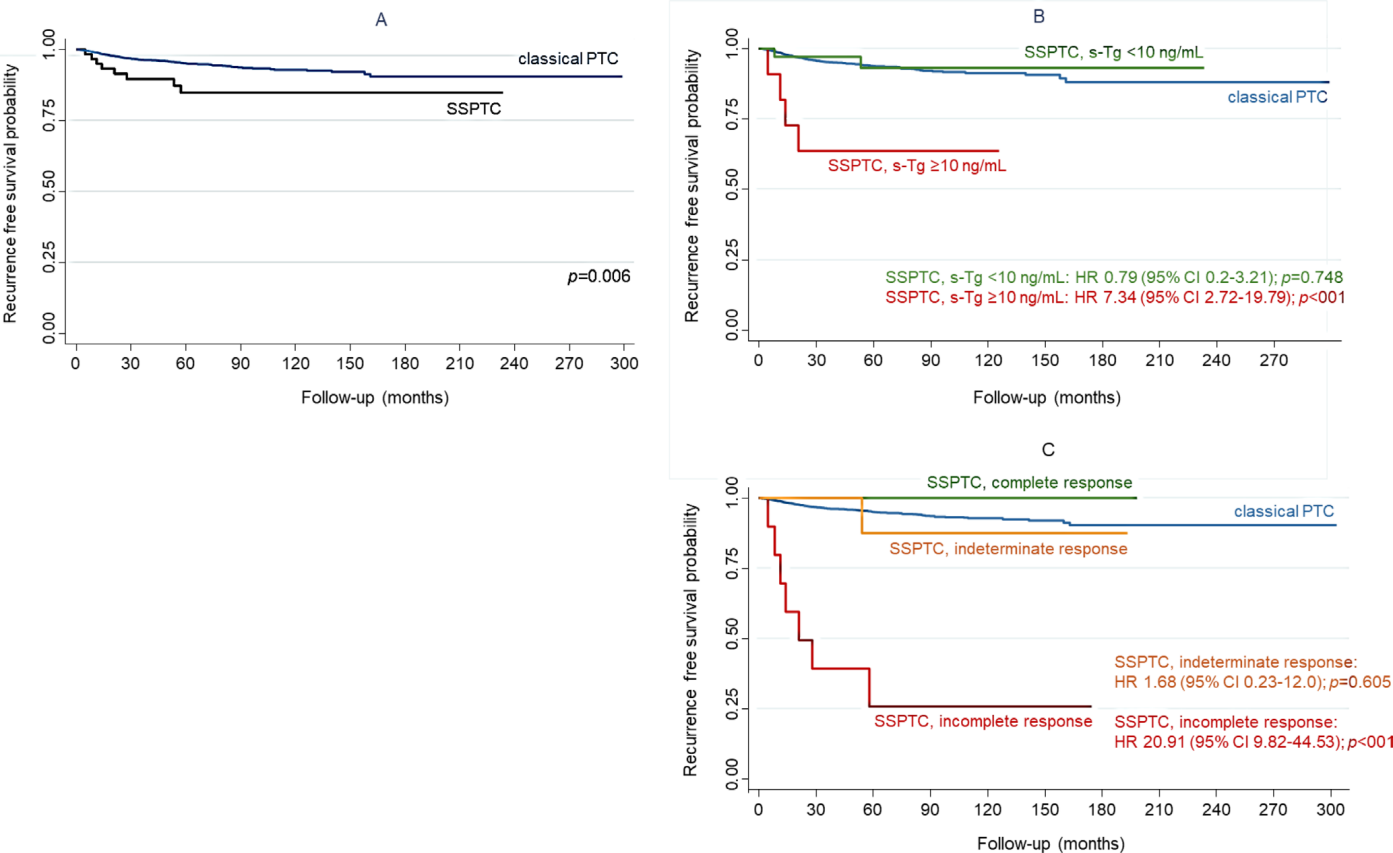
Influence of postoperative stimulated thyroglobulin (s‐Tg) level and response to initial therapy on the probability of recurrence‐free survival in patients treated for solid subtype papillary thyroid carcinoma (SSPTC) and comparison with classical PTC—Kaplan Meier survival based on: (A) histology; (B) postoperative s‐Tg level; and (C) response to initial therapy. [Color figure can be viewed at wileyonlinelibrary.com]

**FIGURE 3:**
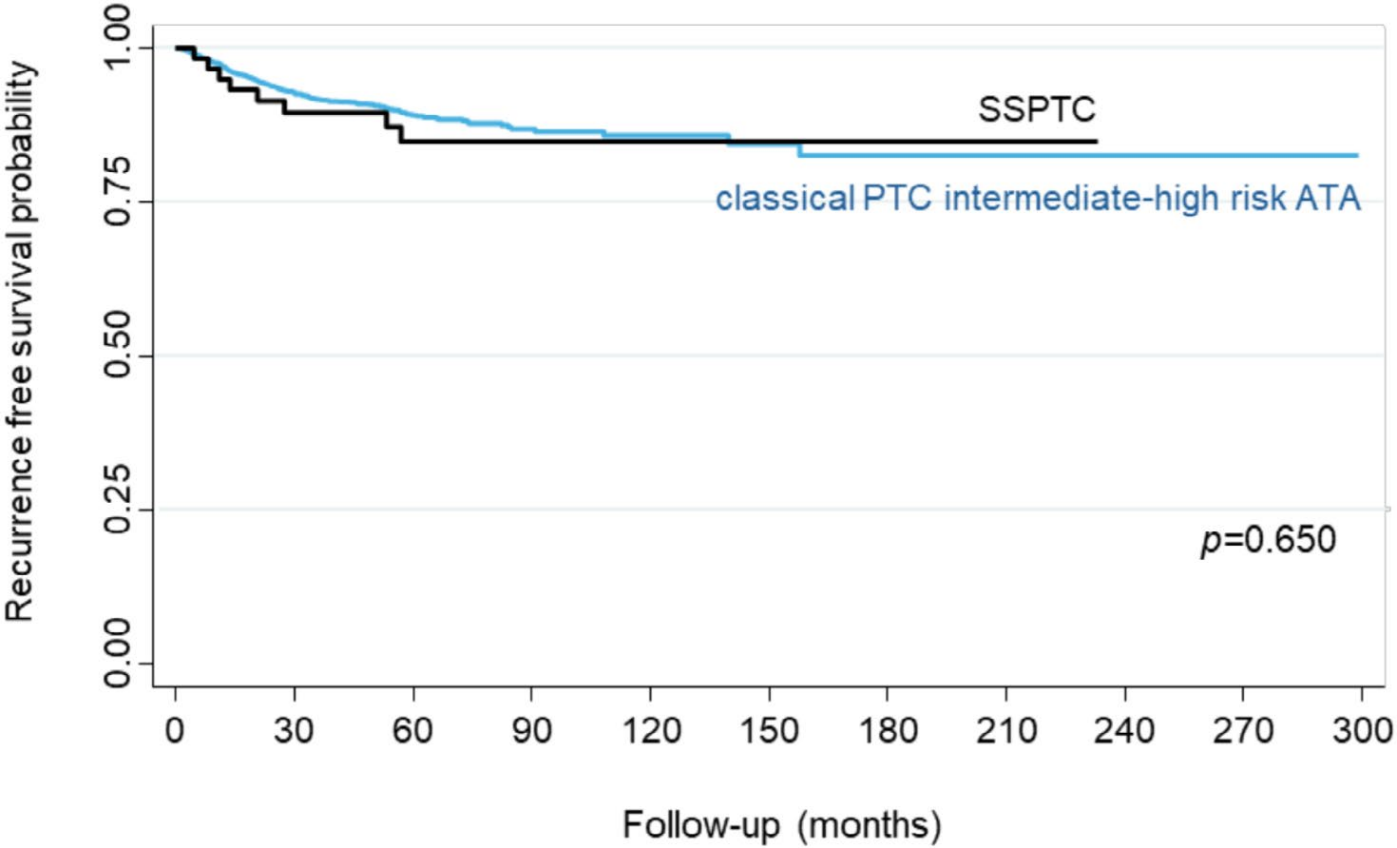
Comparison of the probability of recurrence‐free survival between patients with solid subtype of papillary thyroid carcinoma (SSPTC) and patients with intermediate‐high‐risk classic papillary thyroid carcinoma, according to the American Thyroid Association (ATA) classification. [Color figure can be viewed at wileyonlinelibrary.com]

The probability of 5‐year recurrence‐free survival (RFS) was significantly lower in SSPTC patients with postoperative s‐Tg ≥ 10 ng/mL (63.6%) or who demonstrated an incomplete response after initial therapy (28.6%) compared to cPTC patients (94.9%) (*p* < 0.001). In contrast, SSPTC patients with lower levels of postoperative s‐Tg (< 10 ng/mL) (93.4%) or who demonstrated a complete (100%) or indeterminate (88.2%) response after initial therapy demonstrated RFS risk ratios similar to those of patients with cPTC (94.9%) (*p* < 0.001) (Figure 2). To explore whether the histological subtype itself, independent of other risk factors, impacts RFS, we performed a subgroup analysis comparing SSPTC patients to intermediate‐high risk cPTC patients (Figure 3). This comparison was selected because the cPTC group as a whole contains a much lower proportion of intermediate‐ and high‐risk cases, which could confound the evaluation of RFS. There was no difference in terms of RFS between SSPTC and intermediate‐high risk cPTC.

## 4 Discussion

A few histologic PTC subtypes are listed as aggressive variants and associated with unfavorable outcomes, including tall cell, diffuse sclerosing, columnar cell, solid, and hobnail subtypes [17]. The solid subtype of papillary thyroid carcinoma (SSPTC) is uncommon, and its clinicopathological features and prognostic value remain controversial due to insufficient data [18].

SSPTC was considered a cancer that occurs mainly in children and young adults exposed to radiation, such as survivors of the Chernobyl accident [11]. However, SSPTC can occur sporadically in adult patients who have not been exposed to radiation, with an average age similar to that of classic PTC [14]. In our series, the mean age of patients with SSPTC was 41.1 (±14.2) years, and the female/male ratio was 3/1, data similar to those of patients with cPTC. Only 2 patients had previous exposure to radiation.

In our cohort, SSPTC was associated with cancer recurrence in univariate but not in multivariate Cox regression analysis. SSPTC patients presented larger and more symptomatic tumors (discovered not accidentally) and a higher risk of multifocality, extrathyroidal extension, vascular and lymphatic invasion, pathologically confirmed lymph node metastasis, extra‐nodal extension, and incomplete biochemical response to treatment compared to cPTC, all characteristics related to a higher risk of structural recurrence. Of note, among the 24 patients treated for SSPTC and without any of the other adverse clinicopathological factors, such as extrathyroidal extension, vascular or lymphatic invasion, lymph node metastasis, extra‐nodal extension, and incomplete biochemical response to treatment, there was no cancer recurrence. In a meta‐analysis involving 11 studies with 205 patients, SSPTC was associated with a significantly higher risk of vascular invasion, tumor recurrence, and possibly cancer mortality compared to cPTC. The authors observed that other clinicopathological factors of SSPTC were not statistically different from those of cPTC: sex, age, tumor size, extrathyroidal extension, and lymph node metastasis [19]. Although SSPTC was associated with adverse prognostic features such as tumor size, multifocality, and extrathyroidal extension, it was not an independent predictor of recurrence in multivariate analysis. This suggests that the increased risk of recurrence observed in SSPTC may be mediated by these associated clinicopathological features rather than by the histological subtype itself.

Many authors have confirmed the importance of obtaining postoperative s‐Tg at the time of RAI administration. In general, high postoperative s‐Tg levels (≥ 10 ng/mL) are associated with a higher risk of recurrence [20]. Measurement of postoperative s‐Tg level may be important even in patients with low‐risk to intermediate‐risk PTC who undergo total thyroidectomy and do not receive RAI for remnant ablation, as low postoperative s‐Tg values (< 1 ng/mL) are associated with excellent results in these patients, with a reported recurrence rate below 1% [21]. The integration of postoperative s‐Tg levels into ATA risk categories has an impact in modifying the initial risk estimates [22]. We observed that the probability of cancer recurrence was significantly higher in SSPTC patients with postoperative s‐Tg ≥ 10 ng/mL when compared to patients with SSPTC and lower s‐Tg levels or to patients with cPTC.

Another valuable tool in predicting cancer recurrence is dynamic risk assessment, as additional information obtained during follow‐up can effectively refine initial risk estimates by incorporating treatment response variables (suppressed Tg levels and imaging studies) [16]. In a study recently published by us, 1104 patients with low‐intermediate ATA risk PTCs and s‐Tg levels < 10 ng/mL who demonstrated an excellent response to treatment had a very low recurrence rate (< 0.8%) [23]. In the present study, we observed that none of the patients who demonstrated an excellent response to treatment presented cancer recurrence during follow‐up. In contrast, SSPTC patients who demonstrated an incomplete response after initial therapy had a significantly higher risk of cancer recurrence compared to SSPTC patients who demonstrated a complete or indeterminate response to therapy or to patients with cPTC.

To our knowledge, this is the largest cohort of patients with SSPTC. Furthermore, this is the first study to emphasize the importance of incorporating other important predictive factors of cancer recurrence, such as post‐surgical s‐Tg level and response to initial treatment. Despite this, the sample can still be considered small, due to the rarity of these tumors. Another limitation of this study includes the lack of molecular analysis determination, as some authors suggest that the aggressiveness of SSPTCs may be related to a different molecular pathway than that of cPTCs [24].

In conclusion, our findings indicate that the solid subtype of papillary thyroid carcinoma (SSPTC) is associated with adverse prognostic features, including larger tumor size, multifocality, and extrathyroidal extension, which correlate with a higher risk of cancer recurrence. Although SSPTC itself was not an independent predictor of recurrence in multivariate analysis, its frequent association with high‐risk pathological characteristics supports its classification as an aggressive subtype of PTC. These results highlight the need for a multi‐institutional study with a larger cohort and standardized diagnostic criteria to further elucidate the clinicopathological and molecular features of SSPTC and to optimize risk stratification, therapeutic decision‐making, and long‐term surveillance strategies.

## Disclosure

The authors have nothing to report.

## Conflicts of Interest

The authors declare no conflicts of interest.

## Data Availability

The data that support the findings of this study are available from the corresponding author upon reasonable request.
